# Diagnostic value of multimodal ultrasound imaging in triple-negative breast cancer

**DOI:** 10.3389/fonc.2026.1755646

**Published:** 2026-05-20

**Authors:** Fei Wang, Cong Wang, Jialing Wu

**Affiliations:** 1 Breast Surgery Department of the First Affiliated Hospital of Dalian Medical University, Dalian, China; 2 Ultrasound Department of the First Affiliated Hospital of Dalian Medical University, Dalian, China

**Keywords:** nomogram, SMI, SWE, TNBC, ultrasound

## Abstract

**Objectives:**

This study aimed to evaluate the diagnostic value of multimodal ultrasound imaging in triple-negative breast cancer (TNBC).

**Methods:**

Between January 2023 and January 2025, 274 breast cancers pathologically and molecularly confirmed were enrolled, comprising 88 TNBC and 186 non-TNBC cases. Conventional two-dimensional ultrasound, shear wave elastography, and superb microvascular imaging were performed before biopsy or surgery. Using univariate and multivariate logistic regression analysis, statistically significant variables were screened, and a nomogram was developed.

**Results:**

Multivariate analysis identified microcalcification, stiff rim sign, lesion shape, posterior attenuation, maximum elastic modulus, and vascular index as independent predictors of TNBC. The resulting nomogram demonstrated excellent diagnostic performance, with an area under the curve of 0.965, sensitivity of 0.849, and specificity of 0.966. The optimal diagnostic threshold for the nomogram in predicting TNBC was 0.798.

**Conclusions:**

Multimodal ultrasound is a highly effective tool for the preoperative diagnosis of TNBC.

## Introduction

Breast cancer is currently the most prevalent malignancy globally; however, due to the advancement of early diagnosis modalities and therapeutic strategies, the mortality rate has remained relatively stable. With early detection and timely intervention, the prognosis for patients is considerably improved (1). Triple-negative breast cancer (TNBC) is a subtype characterized by negative expression of estrogen receptor, progesterone receptor, and human epidermal growth factor receptor-2. It exhibits aggressive biological behavior, including high proliferative activity, poor differentiation, and early metastasis, resulting in a suboptimal prognosis (2). Consequently, TNBC remains a focal point and a significant challenge in breast cancer research. Despite lacking effective endocrine and targeted therapies, TNBC exhibits high sensitivity to neoadjuvant chemotherapy, which considerably increases the probability of achieving pathological complete response (3). Therefore, early and accurate diagnosis of TNBC is critical for optimizing treatment strategies, reducing mortality, and improving overall patient prognosis.

The most common imaging modalities for breast cancer diagnosis include mammography, magnetic resonance imaging, and ultrasound, which are complementary and indispensable (4). Breast magnetic resonance imaging offers multi-phasic, multi-sequential imaging with high spatial resolution, resulting in superior sensitivity and specificity for breast diseases. While it is an ideal diagnostic tool, it is relatively expensive and impractical for routine screening (5). Mammography demonstrates high sensitivity for microcalcifications and remains the preferred modality for breast cancer screening. However, its diagnostic value for TNBC is limited by the significantly lower incidence of microcalcifications in this subtype compared with non-TNBC (6). Ultrasound is a non-invasive, accessible, and convenient modality that has excellent soft tissue imaging capabilities. However, a study reported that only 16% of TNBC tumors exhibit typical malignant features on ultrasound, frequently leading to misdiagnosis as benign lesions (7). In recent years, the application of advanced ultrasound technologies, such as shear wave elastography (SWE) and superb microvascular imaging (SMI), has fundamentally enhanced the diagnostic precision for early-stage breast cancer (8).

Research on multimodal ultrasound diagnosis of TNBC is limited, and a diagnostic nomogram has not yet been developed. Therefore, this study aimed to retrospectively analyze the multimodal ultrasound features of TNBC and construct a nomogram to achieve more rapid and accurate preoperative diagnosis, thereby assisting in clinical decision-making.

## Methods

### Ethical approval

The study protocol was approved by the Institutional Review Board of the First Affiliated Hospital of Dalian Medical University (PJ-KS-KY-2023-118) and conducted in accordance with the Declaration of Helsinki. Due to the retrospective nature of the study, the requirement for written informed consent was waived by the approving committee.

### Patients

We retrospectively analyzed the clinical, ultrasound, and pathological data of female patients with breast lesions treated between January 2023 and January 2025. The inclusion criteria were as follows: (1) presence of a solid lesion; (2) no prior radiotherapy or chemotherapy; (3) availability of high-quality two-dimensional ultrasound, SWE, and SMI data; and (4) histopathological and genetic confirmation of the lesion. The exclusion criteria were as follows: (1) cystic nodules; (2) pregnant or lactating women; (3) a history of radiation and chemotherapy; (4) previous breast tumor biopsy, resection, prosthesis implantation, or other surgical procedures; (5) obvious scars adjacent to the breast tumor; and (6) presence of concurrent malignant tumors.

### Ultrasound examination

A Canon Aplio i900 system (Canon Medical Systems Corporation, Tokyo, Japan) equipped with a 10–18 MHZ linear array probe was used. Conventional two-dimensional ultrasound features, including microcalcification, posterior attenuation, aspect ratio, lesion shape, border, and size, were collected. Microcalcification was defined as a calcification with a diameter < 1 mm. Posterior attenuation was defined as a reduction in echoes behind the lesion. The aspect ratio was calculated as the maximum vertical diameter divided by the maximum horizontal diameter. Tumor shape was classified as irregular if more than three lobes were present. An unclear boundary was defined as the lack of a distinct demarcation between the mass and surrounding tissues. Tumor size was recorded as the maximum diameter across all of the planes. For SMI, the color sampling box was adjusted to include the tumor and a 1-cm margin of surrounding tissue. The blood flow velocity was set below 3 cm/s to optimize microvascular visualization while minimizing Doppler artifacts. Following a complete scan, images were frozen and captured at the point of peak vascular density. The region of interest was manually traced to automatically calculate the vascular index (VI). For SWE, the maximum tumor cross-section was identified, maintaining the probe perpendicular to the lesion without external compression. A sampling box of appropriate size was selected; patients were instructed to hold their breath, and images were captured once the color map achieved stable filling. The tumor margin was traced using Q-Box Trace software to obtain the maximum elastic modulus (E-max). All of the images were independently evaluated by two experienced radiologists; any discrepancies were resolved through consensus.

### Statistical analysis

Statistical analyzes were performed using SPSS version 23.0 (IBM Corp., Armonk, NY, USA) and R software (version 3.4.3). Quantitative variables were compared using the Student’s t-test or Wilcoxon rank-sum test, while categorical variables were analyzed using the chi-square test. A p-value < 0.05 was considered to be statistically significant. Least Absolute Shrinkage and Selection Operator (LASSO) logistic regression analysis was performed to select variables. We built a logistic regression model using the stepwise method and established the nomogram using the rms package. Receiver operating characteristic (ROC) curves were plotted using the pROC package, and the optimal cut-off point and area under the curve (AUC) were calculated.

## Results

### Clinical characteristics

**Figure 1:**
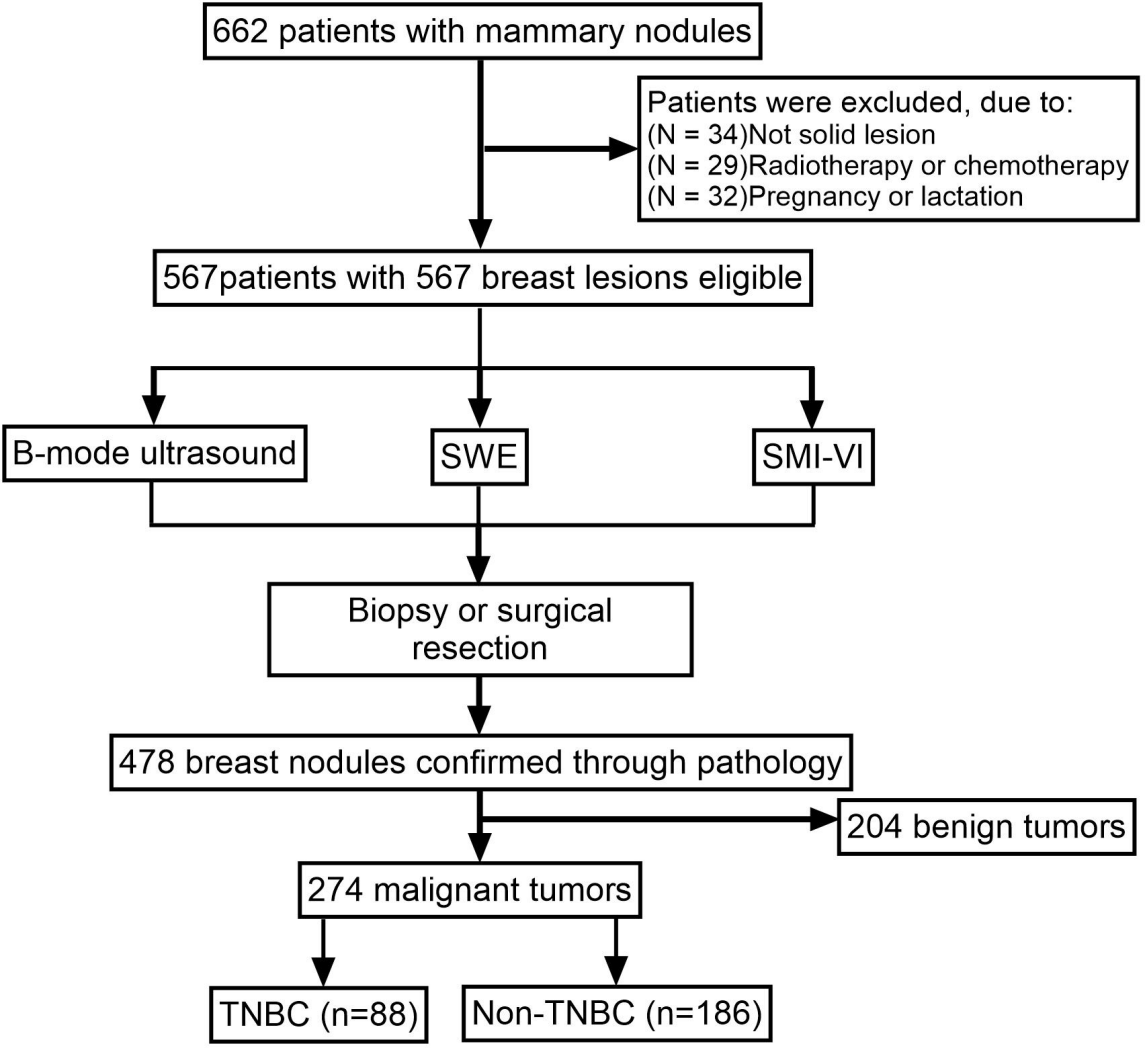
Flowchart of patient enrollment.

**Figure 2:**
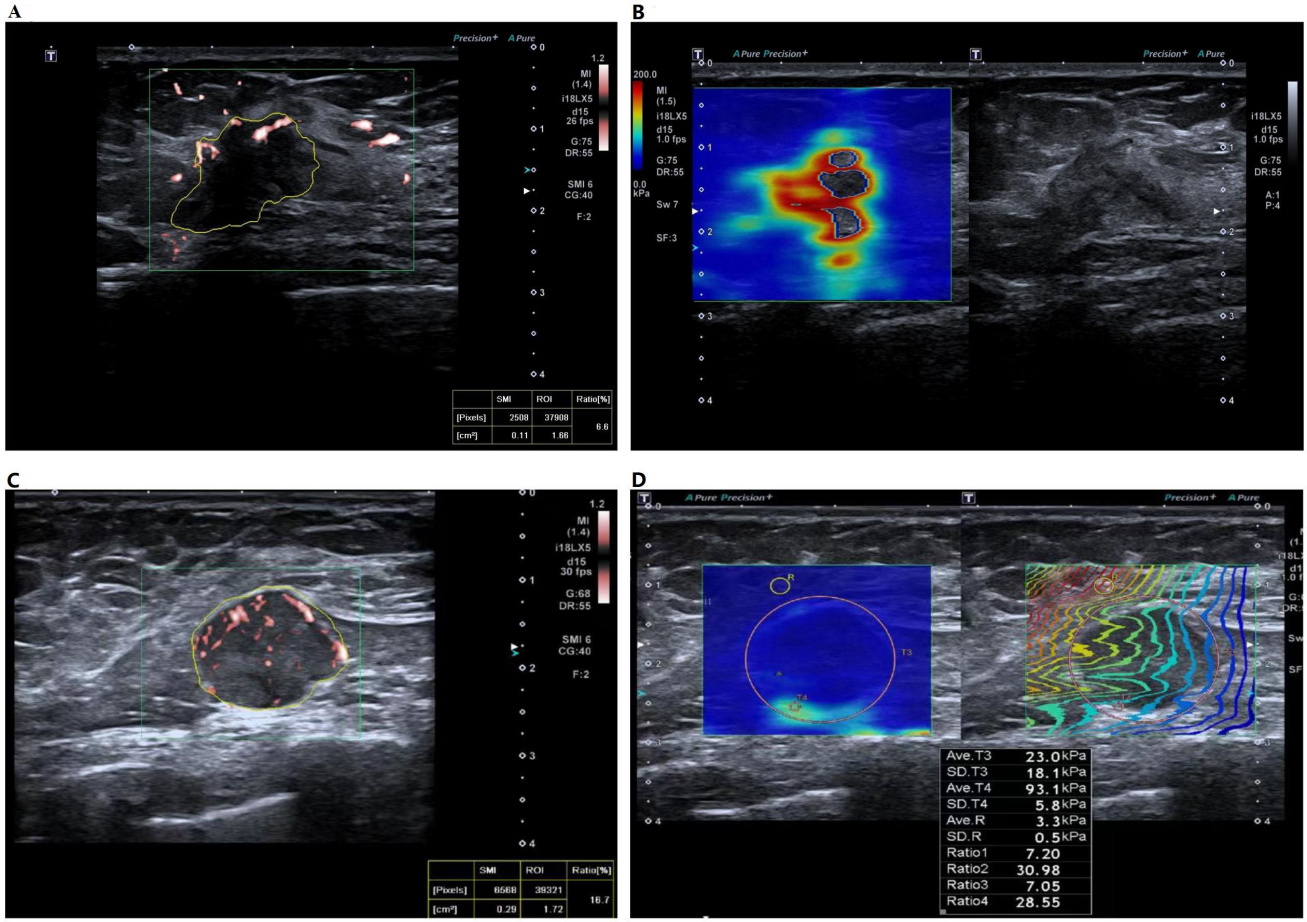
Multimodal ultrasound images of non-TNBC and TNBC cases. **(A)** Non-TNBC showing a vascular index (VI) of 6.6% and **(B)** the stiff rim sign on SWE. **(C)** TNBC showing a VI of 16.7% and **(D)** relatively homogeneous elasticity on SWE.

A total of 274 patients, comprising 88 TNBC and 186 non-TNBC cases, were enrolled in this study. The study flowchart is presented in **Figure 1**. Representative cases of TNBC and non-TNBC, including their respective multimodal ultrasound features, are illustrated in **Figure 2**. **Table 1** summarizes the descriptive statistics and univariate analysis of the multimodal ultrasound features.

**Table 1:** Single factor analysis results of ultrasonic examination parameters.

Variables	TNBC	non-TNBC	Test statistics	*P* value
Total	88	186		
Age(year)			t-test = 1.45	0.149
Mean(SD)	58.5 (9.9)	56.4 (11.9)		
Symptom			Chisquare = 0.01	0.939
No	12 (13.6)	26 (14)		
Yes	76 (86.4)	160 (86)		
Size(mm)			U = 7560	0.309
Median(IQR)	22.5 (17,29.2)	21 (15,31)		
Shape			Chisquare = 42.98	< 0.001
Regular	74 (84.1)	78 (41.9)		
Irregular	14 (15.9)	108 (58.1)		
Boundary			Chisquare = 0.18	0.404
Clear	12(13.6)	76(86.4)		
Unclear	22(11.8)	164(88.2)		
Aspect Ratio			U = 6939	0.042
Median(IQR)	0.7 (0.6,0.8)	0.7 (0.5,0.8)		
Microcalcification			Chisquare = 22.48	< 0.001
Absent	62 (70.5)	74 (39.8)		
Present	26 (29.5)	112 (60.2)		
Posterior echo attenuation			Chisquare = 19.78	< 0.001
No	73 (83)	103 (55.4)		
Yes	15 (17)	83 (44.6)		
VI(%)			U = 3350	< 0.001
Median(IQR)	8.5 (6.5,10.9)	5.6 (3.5,7.3)		
Emax(kPa)			t-test = 11.52	< 0.001
Mean(SD)	95.6 (17)	122.5 (18.6)		
Stiff Rim			Chisquare = 21.52	< 0.001
No	59 (67)	69 (37.1)		
Yes	29 (33)	117 (62.9)		

### Variable selection

**Figure 3:**
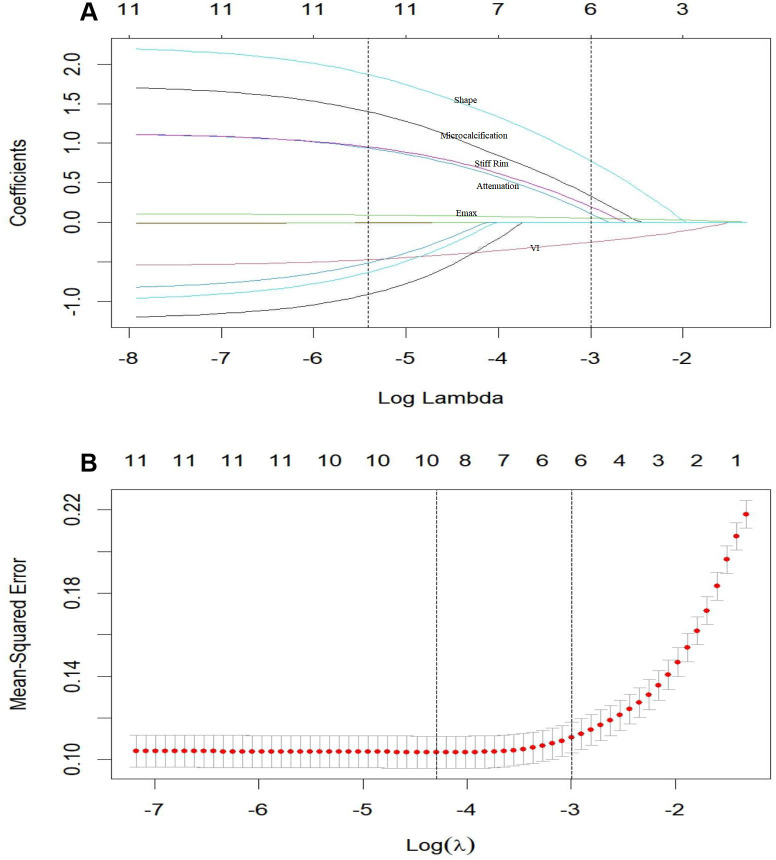
**(A)** LASSO regression path diagram illustrating the selection of the optimal tuning parameter (λ). The iteration process terminated at 75 iterations with a minimum λ value of 0.000274. **(B)** LASSO regression 10-fold cross-validation results. Utilizing the 1-SE criterion, six predictors with non-zero coefficients were identified for inclusion in the final model.

**Figure 4:**
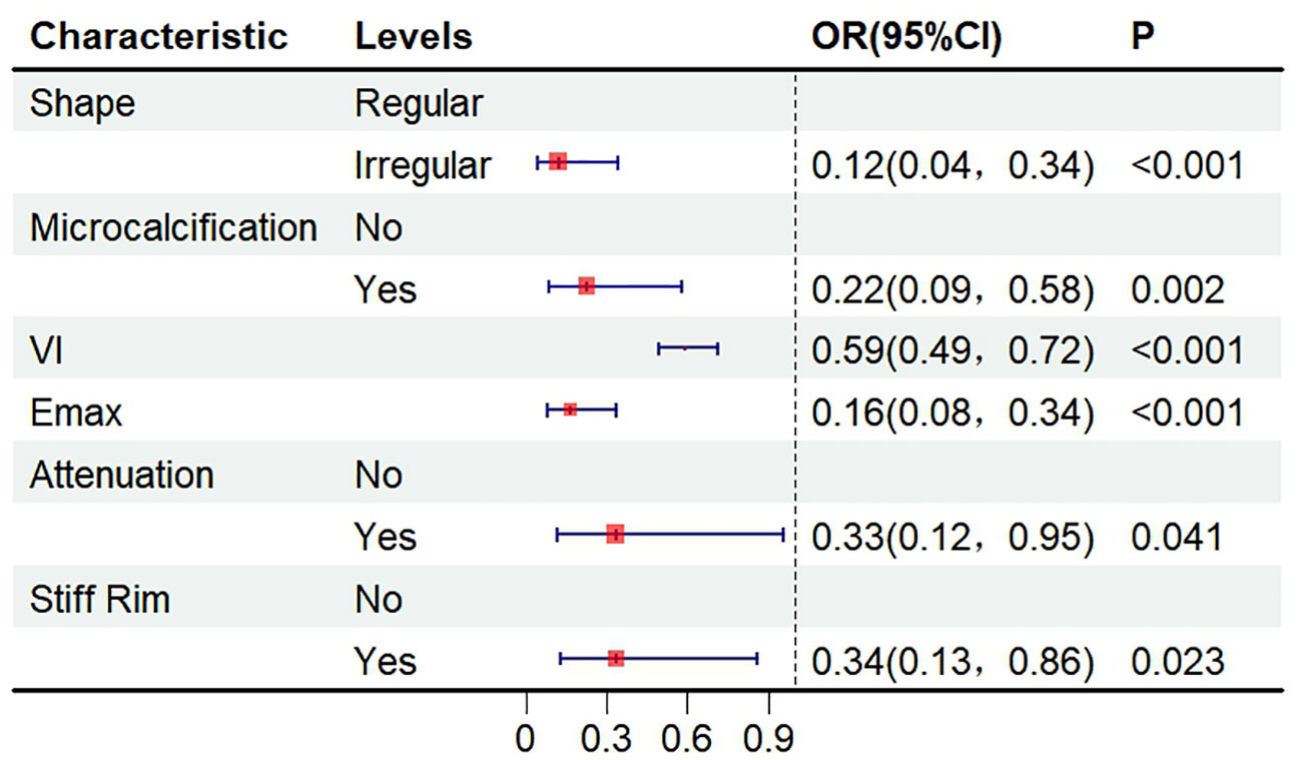
Forest plot of the multivariate logistic regression analysis.

LASSO analysis identified microcalcification, stiff rim sign, lesion shape, posterior attenuation, E-max, and VI as independent predictors of TNBC (**Figure 3**). The results of the multivariate logistic regression analysis are presented in **Figure 4**. Compared with non-TNBC, TNBC was characterized by a regular shape, a low incidence of microcalcifications and posterior attenuation, low hardness, stiff ring sign, and abundant blood flow.

### Development and validation of the predictive model

**Figure 5:**
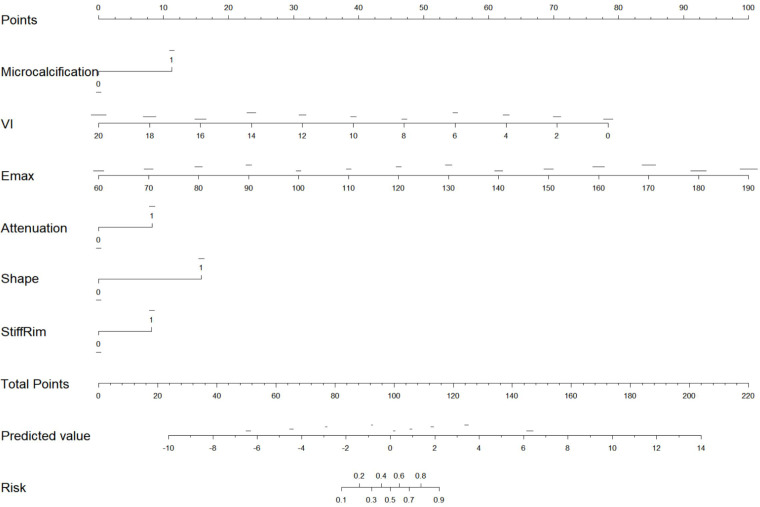
Diagnostic nomogram incorporating two-dimensional ultrasound features, SWE, and VI.

**Figure 6:**
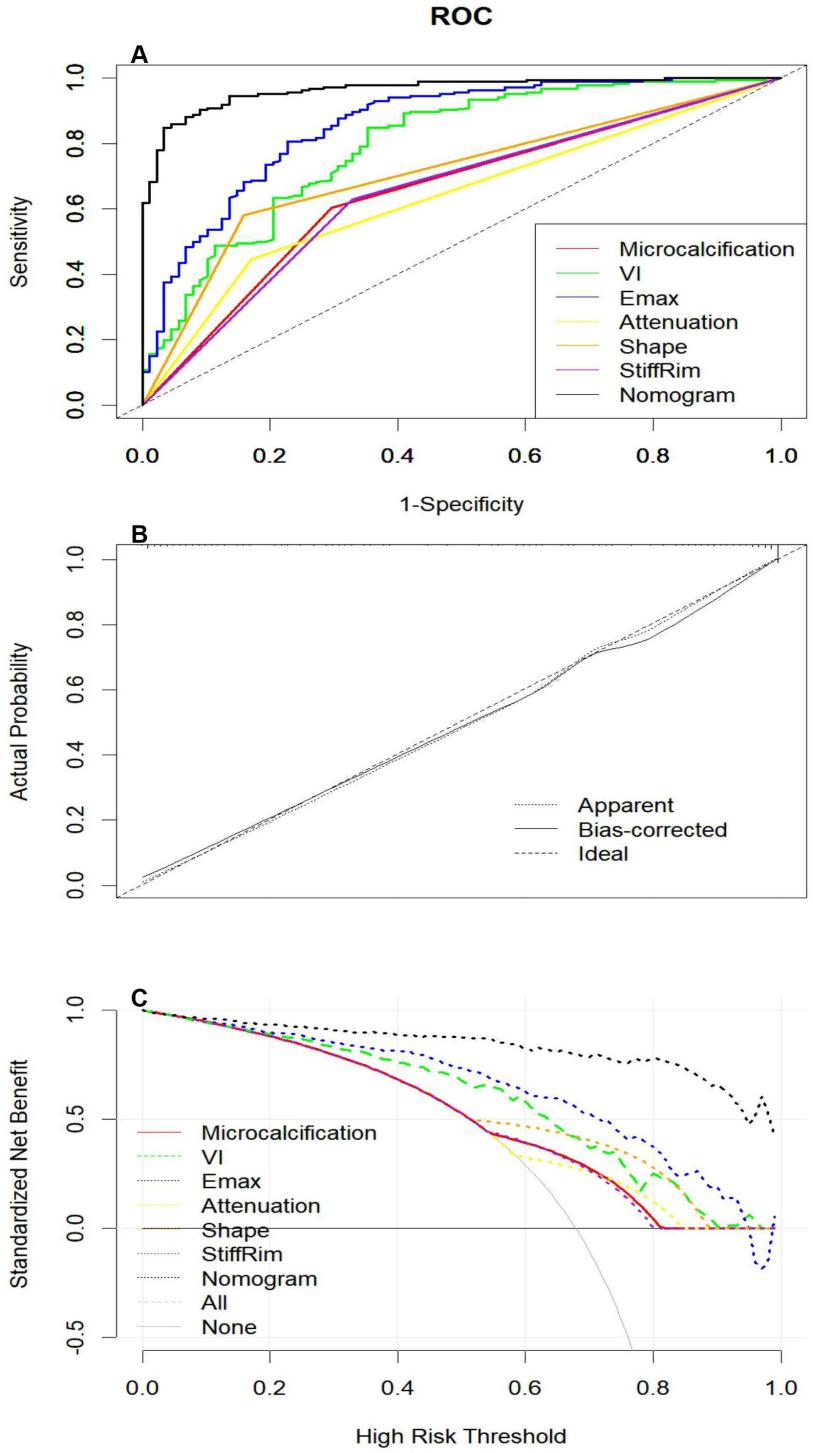
**(A)** Receiver operating characteristic curves comparing multimodal ultrasound parameters with the integrated nomogram. **(B)** Calibration curves of the nomogram. **(C)** Decision curve analysis for the predictive model.

Based on the binary multivariate logistic regression analysis, a nomogram was constructed (**Figure 5**). The ROC curves for the prediction model are shown in **Figure 6A**. Among the individual predictors, E-max demonstrated the highest diagnostic performance with an AUC of 0.858. This was followed by VI (0.795), lesion shape (0.711), microcalcification (0.653), and stiff rim sign (0.65). Posterior attenuation yielded the lowest predictive value, with an AUC of 0.638. The combined multimodal nomogram demonstrated superior diagnostic performance, with an AUC of 0.965, sensitivity of 0.849, and specificity of 0.966. The optimal diagnostic threshold was determined to be 0.798 (**Table 2**). The calibration curve confirmed the nomogram’s high predictive accuracy (**Figure 6B**), while decision curve analysis demonstrated its substantial clinical utility (**Figure 6C**).

**Table 2:** Characteristics of the prediction models.

Variables	Cutoff	AUC	Sensitivity	Specificity	PLR	NLR
Microcalcification	--	0.653	0.602	0.705	2.038	0.565
VI	7.855	0.795	0.648	0.849	4.303	0.415
Emax	106.85	0.858	0.806	0.773	3.548	0.25
Attenuation	--	0.638	0.446	0.83	2.618	0.668
Shape	--	0.711	0.581	0.841	3.65	0.499
Stiff Rim	--	0.65	0.629	0.67	1.909	0.553
Nomogram	0.798	0.965	0.849	0.966	24.918	0.156

PLR, positive likelihood ratio; NLR, negative likelihood ratio; AUC, area under the receiver operating characteristics.

## Discussion

In recent years, the incidence of breast cancer has steadily risen. TNBC is a distinct type of breast cancer that is characterized by unique pathological characteristics. The majority of TNBC cases exhibit low differentiation, highly active cell proliferation, elevated mitotic counts, and high histological grades. Given its aggressive biological behavior and high degree of malignancy, TNBC necessitates rigorous clinical attention (9).

Ultrasound examination is radiation-free, accessible, and comprehensive, making it the preferred diagnostic modality for breast diseases. The differential diagnosis of benign and malignant breast tumors using conventional two-dimensional ultrasound is well-established (10). A previous study showed that on conventional US, TNBC typically presents with micro-lobulated margins, distinct boundaries, and an absence of internal microcalcification (11). Our findings corroborate this, as TNBC frequently demonstrated a regular shape, clear boundaries, lack of posterior attenuation, and an absence of microcalcifications—features that closely mimic the appearance of benign breast tumors.

The aspect ratio of breast masses is a standard descriptor in the BI-RADS classification system; an aspect ratio > 1 is a key malignant feature. While malignant tumors typically demonstrate invasive growth, benign tumors are characterized by expansive growth within a limiting capsule; these divergent biological behaviors result in significantly different aspect ratios (12). However, in the present study, the aspect ratio was not identified as an independent risk factor and was therefore excluded from the final predictive model for distinguishing TNBC from non-TNBC.

Tumor growth, infiltration, and metastasis are driven by neoangiogenesis, which provides the essential microvascular network required for malignant progression (13). Vascular epidermal growth factor can promote angiogenesis, and its expression levels are notably higher in TNBC (14). Based on the Doppler principle, SMI utilizes adaptive image processing to analyze blood flow signals, effectively suppressing motion artifacts and clutter while preserving low-velocity microvascular flow (15). SMI enables the dynamic, multi-angle observation of the entire tumor’s neovascular network without contrast agents, thereby providing a more accurate representation of intratumoral microcirculation (16). Previous studies have indicated that SMI has relatively high sensitivity, specificity, and accuracy in differentiating between benign and malignant breast lesions (17, 18). However, the diagnostic utility of SMI specifically for TNBC has not been previously reported. In this study, the SMI-VI of TNBC was significantly higher than that of non-TNBC.

The occurrence of breast tumors is characterized by complex pathological morphology and histological variations (19). Collagen fibers produced by tumor cells are the primary component of the tumor extracellular matrix, with matrix hardness increasing in proportion to collagen content (20). The tissue hardness of benign and malignant breast lesions is approximately five and eight times higher than that of normal glandular tissue, respectively; this disparity provides the pathological basis for utilizing SWE in differential diagnosis (21, 22). This study demonstrated that TNBC exhibited significantly lower E-max values than non-TNBC, consistent with previous research findings (23). This is potentially attributable to the expansive growth pattern of TNBC; while these tumors exhibit rapid cellular proliferation, their collagen composition is relatively small. Research has shown that breast cancers may exhibit a stiff rim sign on elastographic color maps, representing peritumoral stiffness and the infiltration of malignant cells into surrounding tissues (24). However, our results indicate that the prevalence of the stiff rim sign is significantly lower in TNBC than in non-TNBC. This may be due to the rapid growth rate of TNBC, leading to clinical detection before extensive peritumoral infiltration occurs.

Posterior attenuation of echoes behind breast lesions is typically considered a malignant characteristic. However, the incidence of attenuation in TNBC in this study was significantly lower than in non-TNBC. This may be because non-TNBC possesses a higher collagen content, creating more acoustic impedance interfaces.

In the present study, a multimodal ultrasound nomogram was developed to predict the triple-negative subtype in breast cancer. The model, incorporating two-dimensional ultrasound features, VI, and E-max, showed high sensitivity and specificity. However, several limitations must be acknowledged. First, the lack of an external validation cohort necessitates further research to confirm the model’s reliability and generalizability. Second, the retrospective nature of the study introduces inherent selection bias. Finally, given the relatively small sample size, subsequent large-scale, prospective, multicenter studies are required to further validate these findings.

## Conclusions

In the present study, we developed a multimodal ultrasound nomogram incorporating two-dimensional ultrasound features, SWE, and VI to diagnose TNBC. The proposed model demonstrates excellent diagnostic performance and good clinical usefulness.

## Data Availability

The original contributions presented in the study are included in the article/Supplementary Material. Further inquiries can be directed to the corresponding author.
